# Effects of blood sample handling procedures on measurable interleukin 6 in plasma and serum

**DOI:** 10.1002/jcla.22924

**Published:** 2019-05-26

**Authors:** Yan Gong, Shaocong Liang, Lei Zeng, Yanli Ni, Shaosong Zhou, Xiaopeng Yuan

**Affiliations:** ^1^ Department of Coloproctology Zhujiang Hospital, Southern Medical University GuangZhou China; ^2^ Department of Geriatrics Zhujiang Hospital, Southern Medical University GuangZhou China; ^3^ Department of Laboratory Medicine Zhujiang Hospital, Southern Medical University GuangZhou China

**Keywords:** centrifugal timing, interleukin‐6, sample types, storage time, temperature

## Abstract

**Introduction:**

Interleukin‐6(IL‐6) measurement is used as a biomarker in medical diagnosis, therapy, and prognosis in various diseases. However, several pre‐analytical factors may yield a false IL‐6 result. In this study, we set out to investigate the effects of corrected blood sample handling procedures on measurable IL‐6.

**Method:**

EDTA plasma and serum samples were collected from 45 healthy individuals. The participants were divided into three groups to perform different handling procedures. Different centrifugal timing, storage temperature, and time were executed on the samples. The changed trends of IL‐6 levels were analyzed.

**Results:**

At baseline, while the paired plasma and serum IL‐6 values had a good correlation, the plasma levels were higher than serum. In general, the unseparated EDTA plasma kept steady with time. With the increase in storage temperature and time, a more pronounced rise in unseparated serum IL‐6 was observed. Nevertheless, the samples in Group 3 which centrifuged and separated immediately kept stable after a different temperature and longtime storage.

**Conclusion:**

Sample types, centrifugal timing, storage temperature, and time may affect the IL‐6 levels. A standard blood sample handling procedure should be performed to ensure the accuracy and stability of IL‐6 values.

## 1 INTRODUCTION

Interleukin‐6(IL‐6), a pleiotropic cytokine, is released by a variety of cell types such as immune cells, endothelial cells, and even certain tumor cells.^1^, ^2^ It is a classic proinflammation cytokine which exhibits broad‐ranging biological activities by involved in the intricate cellular communication. These biological actions occur by IL‐6‐mediated activation of several signaling pathways, including immune function, acute phase response, inflammation, metabolism, cell proliferation.^2^, ^3^ IL‐6 is one of the key components involves in the disease pathogenesis. Commonly, IL‐6 acts as a marker for ongoing inflammation while the elevated levels are found in inflammatory‐based diseases, such as infection,^4^ diabetes,^5^ and rheumatoid arthritis.^6^

As the levels of IL‐6 are associated with medical diagnosis, therapy, and prognosis, monitoring the fluctuation may reflect the progression or regression of diseases. Nowadays, remarkable advances in instrument technology have improved the analytical accuracy of cytokines enormously. However, lacking standardized handling procedures in analysis influence the biomarker role of a cytokine. Major errors of the entire diagnostic process are generated within the extra‐analytical phases.^7^ Especially, the pre‐analytical phase may be the source of error in studying cytokines.^8^

Pre‐analytical variables for the test include blood collection tubes, specimen storage temperature and time, plasma or serum separation, and pretreatment steps. Studies have shown pre‐analytical handling steps can influence sample quality and analytical results.^9^, ^10^ Non‐standardization of pre‐analytical procedures may result in inconsistent laboratory results and misleading clinical judgment, thus limiting the application prospects of the assay.

The fact that half‐life of IL‐6 is short, so that the degradation and production may occur simultaneously during storage under unseparated condition. In addition, coagulation processes affect inflammatory activity, resulting in up‐regulation of proinflammatory cytokines in vitro.^11^ Usually, the pre‐analytical periods are varied for many causes. It is not negligible that immune responses, which affected by environmental temperature and storage time, are still ongoing during the period.

Interleukin‐6 is measured through electrochemiluminescence method in our hospital. However, the change of IL‐6 under unseparated condition is poorly understood. Therefore, we examined the effects on concentrations of IL‐6 in serum or plasma under different storage conditions in the present study. This study of the pre‐analytical precautions helps to carry out the correct measurement of this specific marker in clinical laboratory.

## 2 MATERIALS AND METHODS

### 2.1 Collection and processing of whole blood

**Figure 1:**
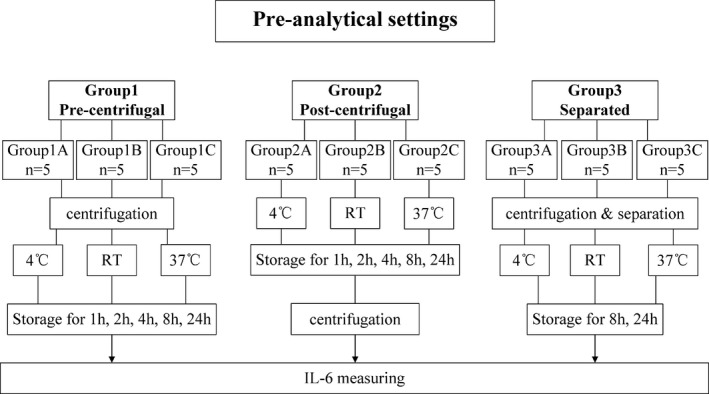
Pre‐analytical settings. Grouping and handling procedures are shown

Whole‐blood samples from 45 health subjects were collected into vacutainer serum tubes (tubes with clot activator) and plasma tubes (tubes with K_2_EDTA; Guangzhou Improve Medical Instruments Co., Ltd.). Immediately after drawing, tubes were gently inverted 10 times. Subsequently, samples were aliquoted into several cryotubes. All study participants were divided into three groups and analyzed further with reference to Figure 1. All samples were centrifuged at 1760 ×*g* for 15 minutes, but centrifugal timing selection was different in three groups. One aliquot from each sample that had been centrifuged and test immediately after blood collection was seen as baseline. The remaining aliquots divided into subgroups and performed different handling procedures. Group 1 were centrifuged and then stored at 4°C room temperature (RT), or 37°C for different periods, while Group 2 was centrifuged after the same storage. Group 3 was centrifuged and then separated immediately before the longtime storage.

### 2.2 IL‐6 measurement

Serum and plasma IL‐6 levels were assayed through the electrochemiluminescence (ECLIA) method on Cobas e601 analyzer (Roche Diagnostics GmbH).

### 2.3 Statistical analysis

Results are expressed as median, as IL‐6 from partial healthy participants were lower than the lower limit of detection. The results below the lowest point of the linear range of the standard curve were assigned detection limit itself and retained for analysis.^12^, ^13^ Serum and plasma of baseline IL‐6 in one individual were compared using Wilcoxon's signed‐rank test and Spearman's rank correlation. The Friedman test was used to evaluate changes in serum or plasma IL‐6 levels over time. A probability (*P*) ≤0.05 was considered statistically significant. Statistical analyses were performed with GraphPad Prism v5.00 software (GraphPad software).

## 3 RESULTS

**Figure 2:**
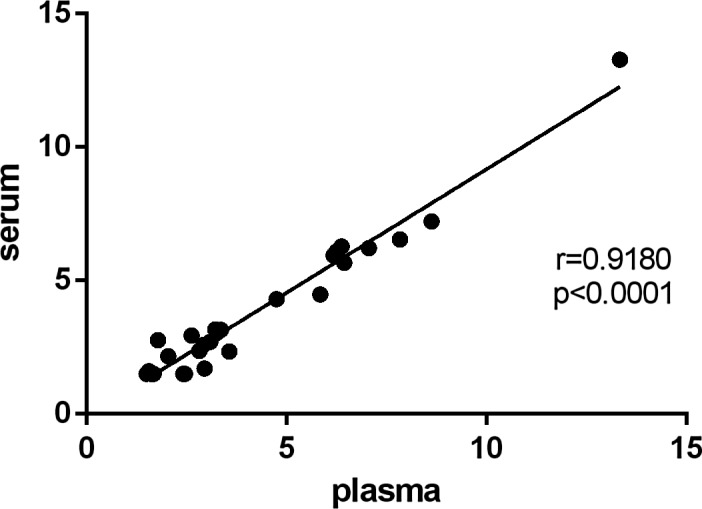
Correlation of the baseline concentrations of IL‐6 between plasma and serum

The median concentrations of IL‐6 in baseline were shown in Table 1. IL‐6 levels were identified to be significantly different between different types of sample, while the levels in plasma were significantly higher compared with serum (*P*＜0.001). In addition, significant correlation was found in the blood between serum and plasma (*P*＜0.0001; Figure 2).

**Table 1:** Baseline IL‐6 levels of Group 1 and Group 2

	Subgroup	n	Age(mean ± SD)	F/M	Type	Median	Min	Max	*P* a
Group 1	Group 1A	5	36.2 ± 13.52	2/3	Plasma	2.46	＜1.50	3.22	
					Serum	1.50	＜1.50	3.17	
	Group 1B	5	39.6 ± 21.00	2/3	Plasma	3.25	＜1.50	3.57	
					Serum	2.71	＜1.50	3.15	
	Group 1C	5	34.0 ± 18.07	2/3	Plasma	1.68	＜1.50	2.63	
					Serum	1.61	＜1.50	2.94	
Group 2	Group 2A	5	26.4 ± 5.41	2/3	Plasma	2.42	1.62	2.93	
					Serum	2.17	＜1.50	2.60	
	Group 2B	5	40.8 ± 4.76	2/3	Plasma	7.84	6.38	13.34	
					Serum	6.54	6.22	13.28	
	Group 2C	5	42.8 ± 8.11	2/3	Plasma	6.18	4.75	6.45	
					Serum	5.67	4.30	6.09	
Total		30	36.6 ± 13.33	2/3	Plasma	3.03	＜1.50	13.34	0.001
					Serum	2.74	＜1.50	13.28	

Abbreviation: F/M, female/male.

a All baseline IL‐6 levels between plasma and serum are compared using Wilcoxon's signed rank test.

**Figure 3:**
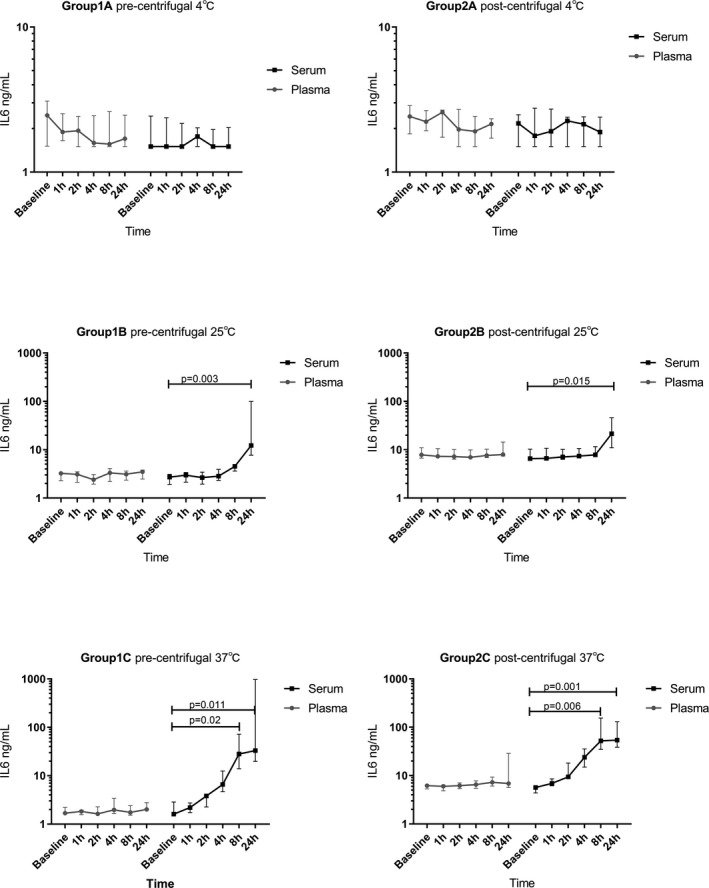
The median (IQR) change trends of IL‐6 in Group 1 and Group 2

Figure 3 shows the changes of median concentrations of IL‐6 in unseparated serum and EDTA plasma after storage at 4°C, room temperature (RT), 37°C from 1 hours to 24 hours. The plasma IL‐6 decreased slightly with time at 4°C, but the decline was not significant. In general, the plasma values were identified to be stable in different temperatures within 24 hours. Similarly, the stable IL‐6 levels are found in serum at 4°C. However, significantly higher concentrations of IL‐6 are found in serum when stored at RT for 24 hours compared to baseline (*P* = 0.003, *P* = 0.015) regardless of centrifugal timing selection (Figure 3. Group 1B and Group 2B). Likewise, the IL‐6 levels are higher than baseline when stored for 8 hours and 24 hours at 37°C (Figure 3. Group 1C and Group 2C).

**Figure 4:**
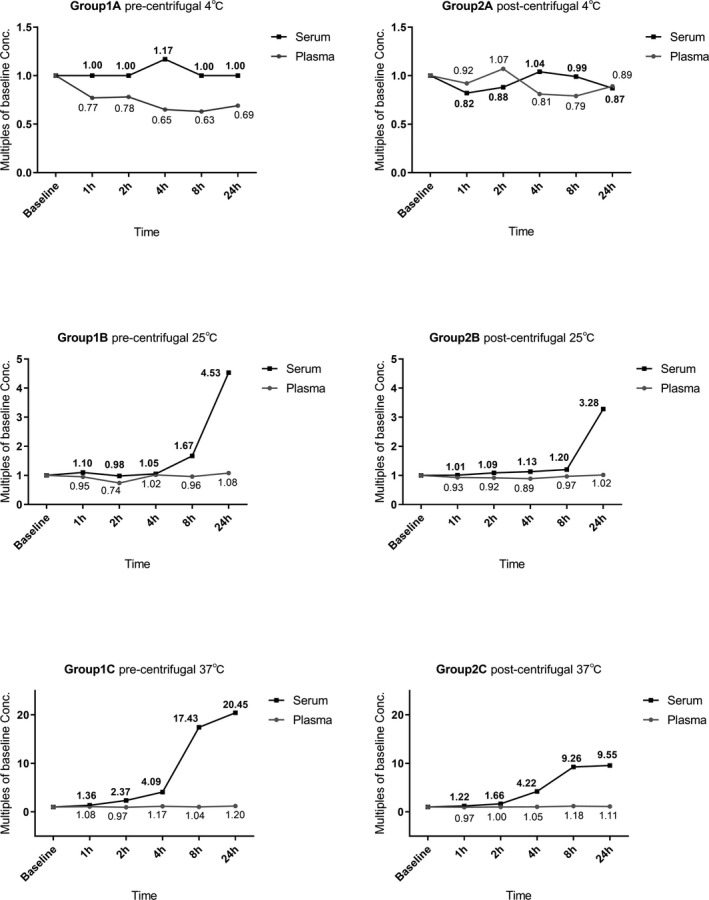
The multiples of baseline Conc. in each detecting point

The change of IL‐6 medians in different blood sample handling procedures, which expressed as multiples of concentrations of baseline, is demonstrated in Figure 4. While the sample was stored at 4°C with pre‐centrifugal, the median of IL‐6 in plasma declined but the trend was stable in serum (Figure 4. Group 1A). Nevertheless, the trends with serum and plasma are uncertain in post‐centrifugal. (Figure 4 Group 2A). Interestingly, though the concentrations of IL‐6 in plasma nearly unchanged, the values in serum rise obviously with time at RT and 37°C. Especially, the higher the temperature is, the earlier and more obvious the increase is. The serum median values increased more than 3‐fold to baseline after storage at RT for 24 hours (Figure 4 Group 1B and Group 2B). Even an increase of more than 4‐fold had shown after storage at 37°C for 4 hours (Figure 4 Group 1C and Group 2C).

The concentration of IL‐6 did not change after the plasma or serum was separated immediately from whole blood with a longtime storage at the same temperature (Table 2).

**Table 2:** Median change trends of IL‐6(ng/L) in separated samples

	Subgroup	n	Age (mean ± SD)	F/M	Temp.	Type	Baseline	8 h	24 h	*P* a
Group 3	Group 3A	5	36.6 ± 16.68	2/3	4℃	Plasma	3.01	2.64	2.73	0.449
						Serum	2.41	2.36	2.60	0.174
	Group 3B	5	49.4 ± 11.84	2/3	RT	Plasma	2.53	2.34	2.92	0.779
						Serum	2.95	3.14	2.74	0.128
	Group 3C	5	41.6 ± 11.93	2/3	37℃	Plasma	2.96	2.57	2.51	0.472
						Serum	2.51	2.62	2.67	0.331

Abbreviations: F/M, female/male; Temp., temperature.

a Friedman test is used in comparing the values.

## 4 DISCUSSION

IL‐6 is a useful diagnostic marker in immune relative diseases. Following the improvement of detection method, the analytical errors of IL‐6 do not influence laboratory diagnostics any more due to the use of ECLIA. However, several pre‐analytical variables, such as specimen types, different temperatures, storage length, or even the centrifugal timing, may still affect the stability of IL‐6 concentrations. Especially, different changes of cytokines may occur during the pre‐analytical period of routine blood samples, from bloods collected to transported and processed.

The potential pre‐analytical variables was evaluated in this study. To be closer to the daily working state, specimens in Group 1 and Group 2 were not isolated from blood cells in our study. The trends of IL‐6 levels were varied during different sample handling procedures. Baseline values showed that the levels of IL‐6 values were affected by specimen types, while it was slightly higher in plasma than in serum. When the samples were stored at four degrees before centrifugation, the plasma values had a slight decrease with time. But in most cases, it remained stable at either RT or warmer condition. Nevertheless, if the specimens were not refrigerated, serum IL‐6 increased with time. At RT, the serum IL‐6 values from health persons kept stable in the first 4 hours. With the increase in storage temperature and time, a more pronounced rise in IL‐6 was observed.

The effect of EDTA is to prevent the blood from clotting in vitro, which is distinct from the coagulant. Through sequestering the calcium ions, EDTA stabilizes the whole blood in fluid form.^14^ Our study on IL‐6 is consistent with the reported that EDTA and cold storage can maintain the stability of cytokines.^14^ However, others indicate that it is not suitable for certain cytokines.^15^ Many studies have shown that cytokine concentrations between different specimen types are different. Some studies demonstrate that basal concentrations are higher in plasma. Some studies, however, are not.^16^, ^17^, ^18^, ^19^ These differences may be attributed to the different sample processing procedures, such as freezing or different testing methods.

There is a dynamic balance between the degradation and production of cytokines in vitro. On the one hand, the half‐life of cytokines is relatively short, and on the other hand, various stimuli lead to the continuous secretion of cytokines by the blood cells.^20^ If one of them is inhibited or promoted, the cytokine level produces a significant change. Nowadays, coagulation has been confirmed to be one of the factors that can induce cytokine release. There is an extensive cross talk between hemostasis and inflammation.^21^, ^22^, ^23^ Inflammation can be modulated by the components of coagulation pathway. Several studies have demonstrated that FVIIa, FXa, and thrombin can active blood cells to produce IL‐6.^24^, ^25^, ^26^ During the blood drawn in serum tubes, the initiation of coagulation pathways is accompanied by the activation of blood cells. We hypothesize that this is a major factor in serum IL‐6 elevation.

As biological reaction requires proper temperature and sufficient time, these may be additional potential influencing factors. In this study, with the storage temperature and length increased, the serum IL‐6 increased obviously. After the same storage period, the IL‐6 elevated more significantly at 37℃ comparing to at RT. Besides, at the same temperature(RT or 37℃), the IL‐6 elevated higher after a longer storage time.

Our study shows that serum IL‐6 increased by more than nine times after storage at 37°C for 8 hours. This range of increase is unacceptable in laboratory analysis. Shockingly, in previous studies, the serum IL‐6 levels can elevate higher, even hundreds of times, after a longtime storage at RT or warmer.^15^, ^27^ Actually, higher and earlier elevated may be found in diseased objects due to the bidirectional relation between inflammation and coagulation. This topic requires a further study.

In practical work, samples usually have no timely detection or even no immediate centrifugation due to various reasons. The centrifugal timing selection was therefore considered in this study to investigate whether it influences IL‐6 change trend. Centrifugation may be another factor to activate immune cells, while the change ratios of serum IL‐6 were higher slightly in pre‐centrifugal. However, similar conditions were not found in plasma samples, while EDTA maybe inhibit certain immune reactions. We also measured the IL‐6 in timely separated plasma and serum, and there are no different of values between storage specimens and baseline. In other words, this confirms the production of cytokines from blood cells in unseparated specimens.

There are potential limitations in this study. First, each subgroup consists of different participants due to the inconvenient in collecting too many blood samples on one person. Second, the sample size is small in each subgroup. Thus, prospective study of a large sample size is needed for validation.

## 5 CONCLUSION

Based on the results of this study, factors that affect changes in cytokine levels are various. Incorrect sample handling procedures may result in false results. In general, unseparated EDTA plasma can keep IL‐6 steady within 24 hours. In not refrigerated conditions, unseparated serum is recommended to measure IL‐6 as soon as possible. Otherwise, the serum separation should be done.

## References

[1] Ho L , Luo S , Lai J . Biological effects of interleukin‐6: Clinical applications in autoimmune diseases and cancers. Biochem Pharmacol. 2015;97:16‐26. 26080005 10.1016/j.bcp.2015.06.009

[2] Ataie‐Kachoie P , Pourgholami MH , Richardson DR , Morris DL . Gene of the month: Interleukin 6 (IL‐6). J Clin Pathol. 2014;67:932‐937. 25031389 10.1136/jclinpath-2014-202493

[3] Rincon M . Interleukin‐6: from an inflammatory marker to a target for inflammatory diseases. Trends Immunol. 2012;33:571‐577. 22883707 10.1016/j.it.2012.07.003

[4] Xia C , Liu Y , Chen Z , Zheng M . Involvement of Interleukin 6 in Hepatitis B Viral Infection. Cell Physiol Biochem. 2015;37:677‐686. 26343270 10.1159/000430386

[5] Pradhan AD , Manson JE , Rifai N , Buring JE , Ridker PM . C‐reactive protein, interleukin 6, and risk of developing type 2 diabetes mellitus. JAMA. 2001;286:327‐334. 11466099 10.1001/jama.286.3.327

[6] Isomäki P , Junttila I , Vidqvist K , Korpela M , Silvennoinen O . The activity of JAK‐STAT pathways in rheumatoid arthritis: constitutive activation of STAT3 correlates with interleukin 6 levels. Rheumatology. 2015;54:1103‐1113. 25406356 10.1093/rheumatology/keu430

[7] Lippi G , Guidi GC , Mattiuzzi C , Plebani M . Preanalytical variability: the dark side of the moon in laboratory testing. Clin Chem Lab Med. 2006;44(4):358‐365. 16599826 10.1515/CCLM.2006.073

[8] Ueland T , Gullestad L , Nymo SH , Yndestad A , Aukrust P , Askevold ET . Inflammatory cytokines as biomarkers in heart failure. Clin Chim Acta. 2015;443:71‐77. 25199849 10.1016/j.cca.2014.09.001

[9] Malentacchi F , Pazzagli M , Simi L , et al. SPIDIA‐RNA: second external quality assessment for the pre‐analytical phase of blood samples used for RNA based analyses. PLoS ONE. 2014;9:e112293. 25384019 10.1371/journal.pone.0112293 PMC4226503

[10] Malentacchi F , Pizzamiglio S , Verderio P , et al. Influence of storage conditions and extraction methods on the quantity and quality of circulating cell‐free DNA (ccfDNA): the SPIDIA‐DNAplas External Quality Assessment experience. Clin Chem Lab Med. 2015;53:1935‐1942. 25883202 10.1515/cclm-2014-1161

[11] Levi M , van der Poll T . Inflammation and coagulation. Crit Care Med. 2010;38:S26‐S34. 20083910 10.1097/CCM.0b013e3181c98d21

[12] Croghan CW , Egeghy PP . Methods of dealing with values below the límit of detection using SAS. Southeastern SAS User Group, 2003.

[13] Whitcomb BW , Schisterman EF . Assays with lower detection limits: implications for epidemiological investigations. Paediatr Perinat Epidemiol. 2008;22:597‐602. 19000298 10.1111/j.1365-3016.2008.00969.x PMC2723785

[14] Banfi G , Salvagno GL , Lippi G . The role of ethylenediamine tetraacetic acid (EDTA) as in vitro anticoagulant for diagnostic purposes. Clinical Chemical. Laboratory Medicine. 2007;45(5):565‐576. 10.1515/CCLM.2007.110 17484616

[15] Lee J , Kim J , Han B , Shin S . Impact of whole‐blood processing conditions on plasma and serum concentrations of cytokines. Biopreserv Biobank. 2016;14:51‐55. 26808439 10.1089/bio.2015.0059

[16] Thavasu PW , Longhurst S , Joel SP , Slevin ML , Balkwill FR . Balkwill FR. Measuring cytokine levels in blood. Importance of anticoagulants, processing, and storage conditions. J Immunol Methods. 1992;153:115‐124. 1381403 10.1016/0022-1759(92)90313-i

[17] Ludviksen JK , Hennø LT , Brekke OL , et al. Elevated cytokine concentrations in serum compared to plasma samples from healthy humans is not explained by in vitro complement activation. Mol Immunol. 2010;47(13):2236–2236.

[18] de Jager W , Bourcier K , Rijkers GT , Prakken BJ , Seyfert‐Margolis V . Prerequisites for cytokine measurements in clinical trials with multiplex immunoassays. BMC Immunol. 2009;10:52. 19785746 10.1186/1471-2172-10-52 PMC2761376

[19] Brøndum L , Sørensen BS , Eriksen JG , et al. An evaluation of multiplex bead‐based analysis of cytokines and soluble proteins in archived lithium heparin plasma, EDTA plasma and serum samples. Scand J Clin Lab Invest. 2016;76:601‐611. 27666533 10.1080/00365513.2016.1230882

[20] Rossol M , Heine H , Meusch U , et al. LPS‐induced cytokine production in human monocytes and macrophages. Crit Rev Immunol. 2011;31:379. 22142165 10.1615/critrevimmunol.v31.i5.20

[21] Levi M , van der Poll T . Coagulation and sepsis. Thromb res. 2017;149:38‐44. 27886531 10.1016/j.thromres.2016.11.007

[22] Chu AJ . Blood coagulation as an intrinsic pathway for proinflammation: a mini review. Inflamm Allergy Drug Targets. 2010;9:32‐44. 19906010 10.2174/187152810791292890

[23] Levi M , van der Poll T , Büller HR . Bidirectional relation between inflammation and coagulation. Circulation. 2004;109:2698‐2704. 15184294 10.1161/01.CIR.0000131660.51520.9A

[24] van der Poll T , de Jonge E , Levi M . Regulatory role of cytokines in disseminated intravascular coagulation. Semin Thromb Hemost. 2001;27:639‐651. 11740687 10.1055/s-2001-18868

[25] de Jonge E , Friederich PW , Vlasuk GP , et al. Activation of coagulation by administration of recombinant factor VIIa elicits interleukin 6 (IL‐6) and IL‐8 release in healthy human subjects. Clin Diagn Lab Immunol. 2003;10:495‐497. 12738659 10.1128/CDLI.10.3.495-497.2003 PMC154959

[26] Sower LE , Froelich CJ , Carney DH , Fenton JN . Klimpel GR. Thrombin induces IL‐6 production in fibroblasts and epithelial cells. Evidence for the involvement of the seven‐transmembrane domain (STD) receptor for alpha‐thrombin. J Immunol. 1995;155:895‐901. 7608566

[27] Skogstrand K , Ekelund CK , Thorsen P , et al. Effects of blood sample handling procedures on measurable inflammatory markers in plasma, serum and dried blood spot samples. J Immunol Methods. 2008;336:78‐84. 18495149 10.1016/j.jim.2008.04.006

